# Probabilistic prediction of segmental body composition in Iranian children and adolescents

**DOI:** 10.1186/s12887-022-03580-z

**Published:** 2022-09-03

**Authors:** Mahsa Rahmani, Arash Ardalan, Mostafa Ghaderi-Zefrehei, Marjan Jeddi, Seyed Taghi Heydari, Mohammad Hossein Dabbaghmanesh

**Affiliations:** 1 Health Policy Research Center, Institute of Health, Shiraz University of Medical Sciences, Shiraz, Iran; 2 Department of Animal Science, Yasouj University, Yasouj, Iran; 3 Endocrinology and Metabolism Research Center, Shiraz University of Medical Sciences, Shiraz, Iran

**Keywords:** Body composition, Adolescent, Obesity, Anthropometry, South of Iran

## Abstract

**Background:**

Adolescents' body composition is considered an important measure to evaluate health status. An examination of any of the segmental compartments by anthropometric indices is a more usable method than direct methods.

**Objectives:**

To propose a method based on the network approach for predicting segmental body composition components in adolescent boys and girls using anthropometric measurements.

**Methods:**

A dual-energy X-ray absorptiometry (DXA) dataset in the south of Iran, including 476 adolescents (235 girls and 241 boys) with a range of 9–18 years, was obtained. Several anthropometric prediction models based on the network approach were fitted to the training dataset (TRD 80%) using bnlearn, an R add-in package. The best fitted models were applied to the validation dataset (VAD 20%) to assess the prediction accuracy.

**Results:**

Present equations consisting of age, weight, height, body mass index (BMI), and hip circumference accounted for 0.85 (*P* < 0.001) of the variability of DXA values in the corresponding age groups of boys. Similarly, reasonable estimates of DXA values could be obtained from age, weight, height, and BMI in girls over 13 years, and from age, weight, height, BMI, and waist circumference in girls under 13 years, respectively, of 0.77 and 0.83 (*P* < 0.001). Correlations between robust Gaussian Bayesian network (RGBN) predictions and DXA measurements were highly significant, averaging 0.87 for boys and 0.82 for girls (*P* < 0.001).

**Conclusions:**

The results revealed that, based on the present study’s predictive models, adolescents' body composition might be estimated by input anthropometric information. Given the flexibility and modeling of the present method to test different motivated hypotheses, its application to body compositional data is highly appealing.

## Introduction

Obesity and overweight in adolescents are serious risk factors for the development of obesity in adulthood. Several clinical studies have discovered strong links between segmental fat mass and health risk, particularly the relationship between trunk fat mass and insulin resistance, dyslipidemia [1], and cardiovascular risk [2] in adolescents, as well as between lean mass and cardio metabolic risk [3]. Adolescence is also a critical period in human life for laying down bone minerals, which increase exponentially in both sexes at this time [4]. Human segmental body composition, which is the distribution of the three parts of body weight (bone, fat, and lean) over the trunk, legs, and arms, is a way to describe what the body is made of and is considered an important measure to evaluate the health status of the human body [5]. Thus, an examination of any of the segmental compartments yields interesting results. Direct body composition measurement methods such as underwater weighing (UWW) and dual-energy X-ray absorptiometry (DXA), though they are accurate and widely acceptable as gold standards in epidemiological studies, are impractical, time-consuming, and expensive in large studies [6, 7]. Moreover, there are physical restrictions on body weight, length, thickness, and width, as well as the type of DXA device. Most obese adults and many obese children are often too wide and too heavy to receive a full-body DXA scan [8]. In clinical and epidemiological studies, simple and practical techniques such as anthropometric-based measurements, which include waist circumference, body mass index (BMI), and skinfold thickness, are the cheapest and most commonly used methods for the estimation of body composition [9]. However, there are some significant drawbacks to each, for instance, BMI tends to overestimate body fat levels in individuals with high muscle mass [10]. For more details, refer to Chung et al. [11]. Statistical methods that use predictive algorithms for assessing body composition have also been developed, which are thought to be more applicable in large studies in non-laboratory settings [5]. Numerous studies have been conducted throughout the world on this topic [5, 12–22]. The literature review revealed that multivariate regression models [12–16], receiver operating characteristic (ROC) curves [17–19], neural networks [21], and Bayesian networks (BN) [5, 22] are among the methods used to predict body composition. In a study to predict body composition, Bayesian networks performed better than linear regression models, and predicted distributions based on this method were close to their published counterparts [22]. In another study, it was suggested that, under certain anthropometric constraints, BN predictions might be used to provide a complementary body composition analysis for large populations [22]. Because of BN’s ability to deal with parametric dimension reduction and uncertainty, it forms the basis of modern methods for assessing many real-world problems, including medical diagnosis and prediction. In the present study, our goal is to predict body compositional measures in adolescent boys and girls in the second-largest country in the Middle East, Iran, using several types of anthropometric data; age, weight, height, waist circumference, hip circumference, and BMI; using a robust Gaussian Bayesian network (RGBN). To our knowledge, until now, no prediction model has been proposed for simultaneously assessing the body composition compartment in the Iranian adolescent population, this research gap led to the present study.

## Methods

The present cross-sectional study was carried out in Kavar, an urban community east of Shiraz, the capital of Fars province in southern Iran, during 2012–2013 [23].

### Subjects

**Fig. 1:**
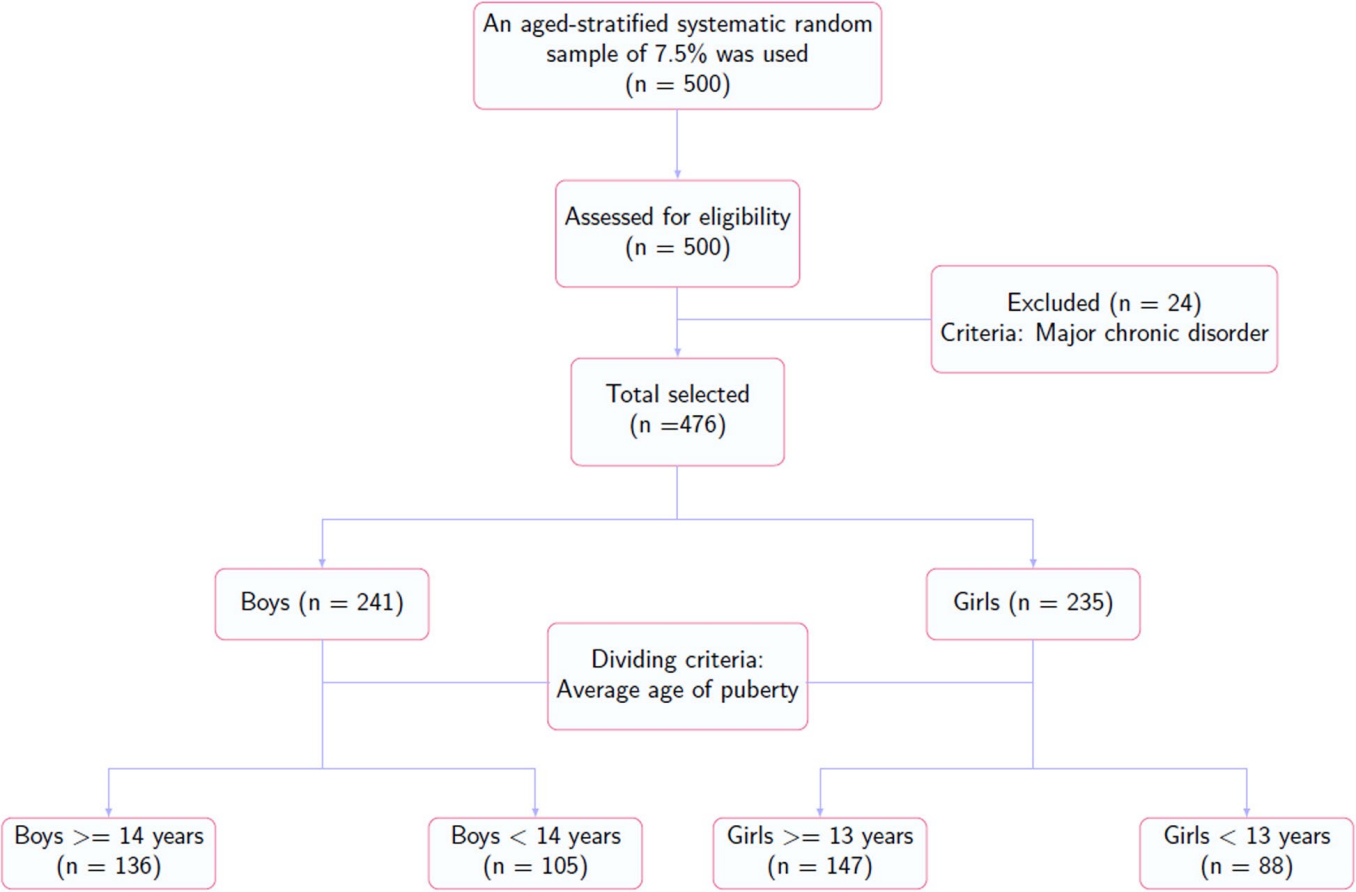
The flowchart of recruitment and research

The participants were girls and boys aged 9–18 years, inhabitants of Kawar, and who were attending elementary to secondary school. An age-stratified systematic random sample of 7.5% was applied, and 500 participants (250 girls and 250 boys) were selected for this study. At each school and for each age group, an alphabetized list of student names was used and a random starting point was picked. Then, every fifth student was selected to take a survey. From them, each participant with a major chronic disorder was excluded, and eventually 476 participants (235 girls and 241 boys) who completed the study were included in the statistical analysis. The participants were divided according to the average age of puberty. As children go through puberty, there are significant effects on body composition. Girls acquire a greater amount of fat mass, and boys gain significantly more fat-free mass, and both genders gain bone mineral density at the highest rate [24, 25]. The cut-off age for girls is ≥ 13 years (88 participants) and for boys it is ≥ 14 years (136 participants). The flowchart of recruitment and research is shown in Fig. 1.

#### Anthropometric measures

Weight and height were measured by a training nurse, weight using the standard scale closest to 0.1 kg (Seca, Germany) and height at the nearest 0.5 cm using a wall-mounted meter with the participant wearing light clothing and no shoes. BMI was calculated according to this formula: BMI (kg/m^2^) = weight (kg) / (height (m))^2^. Also, we measured the waist circumference to the nearest 0.1 cm just above the patient’s uppermost lateral border of the right ilium.

#### Body composition measures

The Hologic system (Discovery QDR, USA) was used to measure BMC (in gram), bone area (in square centimeter), BMD (in gram per square centimeter), lean mass, and fat mass (in gram). Bone mineral density was measured in the total body (without the head), lumbar spine, and left femoral neck by a single experienced technician. Lean mass and fat mass were measured in the total body as, well as in specific body compartments, such as the trunk, legs, and arms. Densitometric studies were done with the participant wearing special clothing and no footwear. To eliminate physiological lumbar lordosis during measurement of the lumbar spine, we elevated the participants’ knees while they were in a supine position. In accordance with international standards, all measurements of the femur were done on the left femur at the position of internal rotation. During DXA measurements, the position, size, and location of the region of interest were the same for all participants. Scanner stability was checked throughout the course of the study with plots of daily spine phantom scans. Based on preliminary measurements in ten children, the coefficients of variation in our laboratory were 1% for the total body, 0.51% for the lumbar spine, and 2.4% for the femoral neck [23].

### Statistical analysis

The statistical method that we used in the present study was based on BNs [26–28] which is a graph-based method that represents complex probabilistic interactions and causality relationships between variables [29]. In plain language, RGBN was used to predict segmental body composition compartments by simple anthropometric indices according to the method provided by Kenett et al. [30]. According to the cut-off age, there are 4 groups under study: girls over 13 years (147 participants); girls under 13 years (88 participants); boys over 14 years (136 participants); and boys under 14 years (105 participants). Each group was divided into a training dataset (TRD: 80%) and a validation dataset (VAD: 20%). For each group, different subsets of predictor variables were considered to construct the Gaussian Bayesian network (GBN) [31, 32], which was based on the assumption that the variables followed a normal distribution. To learn the network structure related to each subset of predictor variables, 12 structure-learning algorithms (4 constraint-based algorithms [33], two score-based algorithms [34] and two hybrid algorithms [35] with different parameters) that limited the network arcs according to prior knowledge were used. To run the model, an add-in package, “bnlearn in R [36] was used. After identifying the network structure with the stated algorithms, to create an RGBN from all possible edges between the predictor variables and the response variables, only the edges that appeared in at least seven algorithms were present in the RGBN. The Bootstrap method was used to evaluate the robustness of the created structures. In this way, by generating 500 random subsets of data related to each group and each with 500 observations, the occurrence ratio of each edge in the bootstrap iterations was measured. Those that did not have the minimum occurrence rate (70%) were removed from the network. The quality of RGBNs made for each subset of predictors variables was evaluated with four criteria:$\left|Bias\right|={\sum }_{v=1}^{p}{\sum }_{i=1}^{n}{B}_{{i}^{v}}$,$SD={\sum }_{v=1}^{p}{\sigma }^{v}$,$SEP= {\sum }_{v=1}^{p}({\sum }_{i=1}^{n}\sqrt{{\left|{B}_{{i}^{v}}\right|}^{2}+{({\sigma }^{v})}^{2}})$, and the number of edges. Following the selection of the RGBNs, the linear model corresponding to each response variable was extracted from the networks. Prediction of body composition should be done hierarchically. The variable that was the offspring^1^ node of anthropometric indices was predicted first, and then the offspring of this variable were estimated using the predicted values. Thus, all body composition variables were predicted. The normal distribution of data was checked using the Kolmogorov–Smirnov test and confirmed by a visual inspection of the histograms and Q-Q plots. Skewed variables were log transformed. Student t-tests were used to compare sex differences in all variables' means. Results with a p-value < 0.05 were considered statistically significant. The statistical power of the study was assessed by the "pwr" package in R [37] and for all of the analyses, it was greater than 0.95.

## Results

Except for weight and body mass index, all the anthropometric variables were significantly different for girls compared with boys (Table 1). In addition, for DXA body composition compartments, except for the trunk BMC, all the variables differed significantly between boys and girls.

**Table 1:** Summary statistics for anthropometric indices and body composition variables, including mean, skewness, standard deviation (SD), and quartiles, as well as their units, are presented by gender

		Mean		Quartiles		SD	Skewness	
Variables	Gender		25%	50%	75%			*P-value
Trunk Fat (kg)	Boys	1.47	1.26	1.42	1.62	0.27	0.77	< 0.0001
	Girls	4.41	2.84	4.07	5.79	1.88	0.48	
Trunk Lean (kg)	Boys	15.29	11.30	14.81	19.29	4.26	0.29	< 0.0001
	Girls	13.65	11.47	14.25	15.79	2.93	-0.23	
Trunk Bone (kg)	Boys	0.37	0.26	0.34	0.48	0.13	0.53	0.244
	Girls	0.36	0.27	0.37	0.44	0.63	0.22	
Leg Fat (kg)	Boys	1.56	1.32	1.53	1.74	0.29	0.44	< 0.0001
	Girls	4.96	3.44	4.82	6.39	1.76	0.17	
Leg Lean (kg)	Boys	11.73	8.29	11.13	15.23	3.75	0.19	< 0.0001
	Girls	9.32	7.66	9.60	10.70	2.01	-0.09	
Leg Bone (kg)	Boys	0.55	0.38	0.52	0.71	0.19	0.40	< 0.0001
	Girls	0.48	0.38	0.49	0.57	0.11	0.05	
Arm Fat (kg)	Boys	0.79	0.67	0.77	0.88	0.17	0.83	< 0.0001
	Girls	1.19	0.65	1.09	1.62	0.63	0.54	
Arm Lean (kg)	Boys	3.59	2.33	3.34	4.77	1.42	0.39	< 0.0001
	Girls	2.72	2.28	2.82	3.22	0.74	-0.37	
Arm Bone (kg)	Boys	0.19	0.37	0.44	0.53	0.09	0.21	0.014
	Girls	0.16	0.11	0.16	0.20	0.05	-0.09	
Age (year)	Boys	14.1	2.5	11.6	13.9	15.7	-0.07	0.95
	Girls	13.8	2.8	11.3	13.60	16.1	0.13	
Weight (kg)	Boys	41.6	31.0	40.0	51.8	12.0	0.38	0.098
	Girls	40.2	31.0	40.0	48.0	10.0	0.10	
Height (cm)	Boys	155.2	142.3	154.0	169.8	15.7	-0.08	< 0.0001
	Girls	150.6	142.0	153.0	159.0	11.0	-0.65	
BMI (*kg/m*^2^)	Boys	16.8	15.1	16.6	18.3	2.3	0.69	0.224
	Girls	17.5	15.4	17.4	19.4	2.7	0.48	
Waist Circumference (cm)	Boys	65.6	58.0	65.0	72.0	9.1	0.28	0.010
	Girls	68.6	63.0	69.0	75.0	9.0	0.08	
Hip Circumference (cm)	Boys	78.6	71.8	78.0	85.0	8.6	0.19	0.002
	Girls	82.0	75.0	82.0	89.0	9.1	0.00	

Note: *P-values for boys vs. girls (Student t test)

**Fig. 2:**
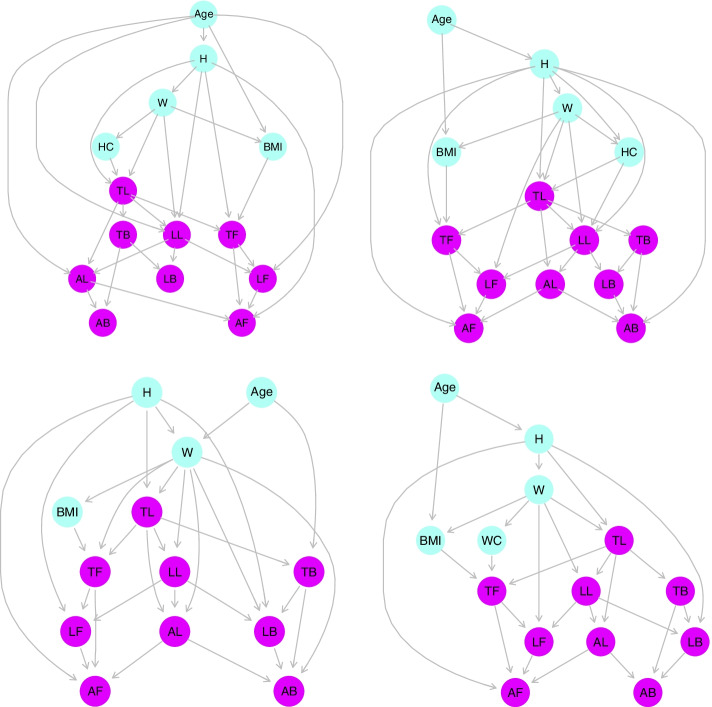
Top: Structures of the chosen RGBNs for boys over 14 years (left) and boys under 14 (right). Bottom: Structures of the chosen RGBNs for girls over 13 years (left) and girls under 13 years (right). W: weight, H: height, WC: waist circumference, HC: hip circumference, TF: trunk fat mass, TL: trunk lean mass, TB: trunk BMC, LF: leg fat mass, LL: leg lean mass, LB: leg BMC, AF: arm fat mass, AL: arm lean mass, AB: arm BMC

Among all the fitted models, including the multivariate regression model with all mentioned anthropometric indices and high parametric dimension, we selected those models that had a small number of parameters with the lowest SEP in the validation data set; the best models were as follows: for boys over 14 years with bias = 4.09, SD = 4.46 and SEP = 6.38; for boys under 14 years with bias = 3.29, SD = 4.63 and SEP = 6.02; for girls over 13 years with bias = 4.02, SD = 5.66 and SEP = 7.35; for girls under 13 years old with bias = 4.95, SD = 5.14 and SEP = 7.76 (Table 2). All direct connections in the final structure of RGBNs were significant (*P* < 0.001) (Fig. 2).

**Table 2:** Some RGBNs we explored by applying RGBNs to all groups under study

				Train Errors				Test Errors		
Groups	BNs	Covariables	N.arcs	|Bias|	SD	SEP		|Bias|	SD	SEP
Boys over 14 years	1	Age-W–H-BMI-WC	25	3.41	4.52	6.05		4.00	4.52	6.39
	2*	**Age-W–H-BMI-HC**	**26**	**3.35**	**4.46**	**5.92**		**4.09**	**4.46**	**6.38**
	3	Age-BMI-WC-HC	25	3.93	5.20	6.90		4.86	5.20	7.54
Boys under 14 years	1	Age-W–H-BMI-WC	24	3.23	4.29	5.72		3.35	4.63	6.05
	2*	**Age-W–H-BMI-HC**	**26**	**3.24**	**4.27**	**5.71**		**3.29**	**4.63**	**6.02**
	3	Age-BMI-WC-HC	21	3.83	5.03	6.71		3.86	5.73	7.25
Girls over 13 years	1	Age-W–H-BMI-WC	27	3.92	5.66	7.29		4.02	5.66	7.35
	2	Age-W–H-BMI-HC	27	3.92	5.66	7.29		4.02	5.66	7.35
	3*	**Age-W–H-BMI**	**27**	**3.92**	**5.66**	**7.29**		**4.02**	**5.66**	**7.35**
Girls under 13 years	1*	**Age-W–H-BMI-WC**	**23**	**3.77**	**4.86**	**6.49**		**4.95**	**5.14**	**7.76**
	2	Age-W–H-BMI-HC	23	3.73	4.81	6.44		5.03	5.13	7.80
	3	Age-W–H-BMI	22	3.82	4.93	6.56		4.97	5.20	7.84

Note: The selected model for interpretation is shown in bold and with a “*”. *W* Weight, *H* Height, *WC* Waist circumference, *HC* Hip circumference

All estimated regression models with standardized coefficients were extracted from RGBNs by considering the parents of each node as predictors of these linear models. In addition, the proportion of variance explained by these models is given for each variable. Predictive models were able to describe the variations in each response variable, which averaged 0.85 for all groups (Table 3).

**Table 3:** Prediction models and proportion of variance explained for each of the nine response variables by the respective parents within the chosen RGBNs

	Models		Explained variance	
Variables	Boys over 14 years	Boys under 14 years	Boys over 14 years	Boys under 14 years
TL	0.66 W + 0.19H + 0.18HC	0.30 W + 0.55H + 0.13 HC	0.88	0.90
TF	1.29BMI + 0.47H − 0.74TL	0.82BMI + 0.45H − 0.37TL	0.76	0.75
TB	0.88TL	0.89TL	0.77	0.82
LL	0.48TL + 0.27 W + 0.21H	0.50TL + 0.26H + 0.33 W − 0.12HC	0.84	0.93
LF	0.91TF − 0.11Age	0.79TF + 0.35 W	0.82	0.81
LB	0.51 TB + 0.41LL	0.63LL + 0.34 TB	0.89	0.95
AF	0.45TL + 0.42LL + 0.11Age	0.81LL	0.84	0.84
AL	0.42TF + 0.28AL + 0.49LF − 0.19H	0.45TF + 0.44LF + 0.49AL − 0.45H	0.87	0.86
AB	0.76AL + 0.19 TB	0.76AL + 0.34LB − 0.19 TB	0.95	0.93
	Girls over 13 years	Girls under 13 years	Girls over 13 years	Girls under 13 years
TL	0.81 W + 0.13H	0.70 W + 0.31H	0.80	0.91
TF	0.34BMI + 0.99 W − 0.33TL	0.68BMI + 0.21WC + 0.19TL	0.80	0.74
TB	0.66TL + 0.29Age	0.89TL	0.64	0.80
LL	0.66TL + 0.31 W	0.78TL + 0.20 W	0.82	0.90
LF	0.80TF − 0.15H	0.60TF − 0.30LL + 0.68 W	0.81	0.88
LB	0.51 TB + 0.22LL + 0.14 W + 0.19H	0.63LL + 0.41 TB + 0.21H	0.69	0.88
AF	0.43LL + 0.23 W	0.87LL	0.62	0.68
AL	0.42TF + 0.28AL + 0.49LF − 0.19H	0.61LF + 0.63AL − 0.24H	0.91	0.84
AB	0.59AL + 0.30 TB + 0.28LB − 0.15 W	0.74AL + 0.26LB	0.81	0.81

Note: *W* Weight, *H* Height, *WC* Waist circumference, *HC* Hip circumference, *TF* Trunk fat mass, *TL* Trunk lean mass, *TB* Trunk BMC, *LF* Leg fat mass, *LL* Leg lean mass, *LB* Leg BMC, *AF* Arm fat mass, *AL* Arm lean mass, *AB* Arm BMC

**Fig. 3:**
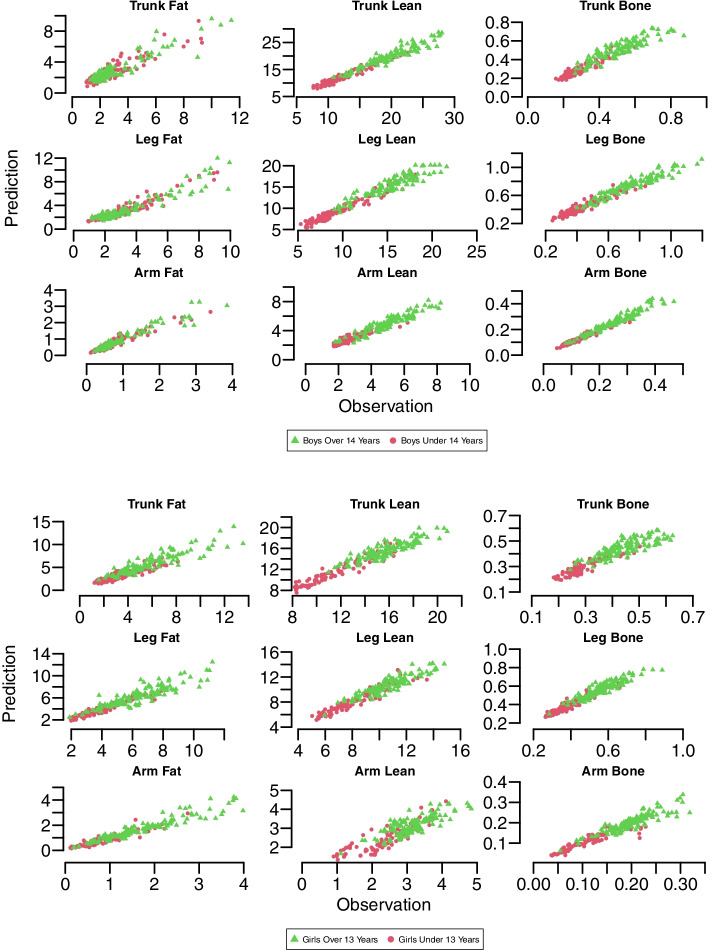
Scatter plots of RGBN prediction model for nine segmental compartments in relation to their DXA observation counterparts

The validation scores for the prediction models as derived from the RGBN method. For the prediction of body composition variables, the maximum SEP value was 1.28 kg for both boys and girls. The corresponding values varied from 0.65 to 0.93 for both sexes. According to the MAPE score, it was possible to estimate the segmental fat-free mass components (lean and BMC) with a maximum difference of ± 9.48% and ± 14.97% of DXA values for boys and girls, respectively. Segmental fat mass could be estimated with less accuracy than other components, with a maximum difference of ± 19.43% and ± 18.97% of DXA values for boys and girls, respectively (Table 4). Segmental body compositions were well predicted overall, even if some bias arose in particular areas, especially for fat masses. Generally, RGBN’s prediction had a fairly strong correlation with DXA measurements (Fig. 3).

**Table 4:** Quality of fit between nine segmental compartments as derived by DXA with their counterparts predicted by the RGBN

Compartments	Boys over 14 years			Boys under 14 years		
	MAPE	SEP	R^2^	MAPE	SEP	R^2^
TF	18.04	0.82	0.82	19.43	0.73	0.81
TL	4.88	1.28	0.90	5.33	0.78	0.93
TB	9.38	0.06	0.78	8.69	0.03	0.84
LF	17.49	0.77	0.89	13.97	0.54	0.91
LL	5.93	1.10	0.84	5.65	0.67	0.94
LB	5.84	0.06	0.89	7.26	0.04	0.90
AF	13.93	0.21	0.90	17.91	0.18	0.88
AL	9.01	0.47	0.88	9.48	0.35	0.83
AB	6.06	0.02	0.94	7.44	0.01	0.93
	Girls over 13 years			Girls over 13 years		
	MAPE	SEP	R^2^	MAPE	SEP	R^2^
TF	14.27	1.06	0.81	17.34	0.76	0.74
TL	4.97	0.93	0.79	4.97	0.76	0.91
TB	9.16	0.05	0.65	8.51	0.03	0.81
LF	10.84	0.87	0.82	18.96	0.43	0.90
LL	5.04	0.67	0.83	4.52	0.60	0.91
LB	5.03	0.04	0.84	5.87	0.03	0.90
AF	12.37	0.28	0.90	18.97	0.19	0.88
AL	10.32	0.37	0.65	14.97	0.38	0.76
AB	8.14	0.02	0.82	11.12	0.02	0.78

Note: *TF* Trunk fat mass, *TL* Trunk lean mass, *TB* Trunk BMC, *LF* Leg fat mass, *LL* Leg lean mass, *LB* Leg BMC, *AF* Arm fat mass, *AL* Arm lean mass, *AB* Arm BMC

**Fig. 4:**
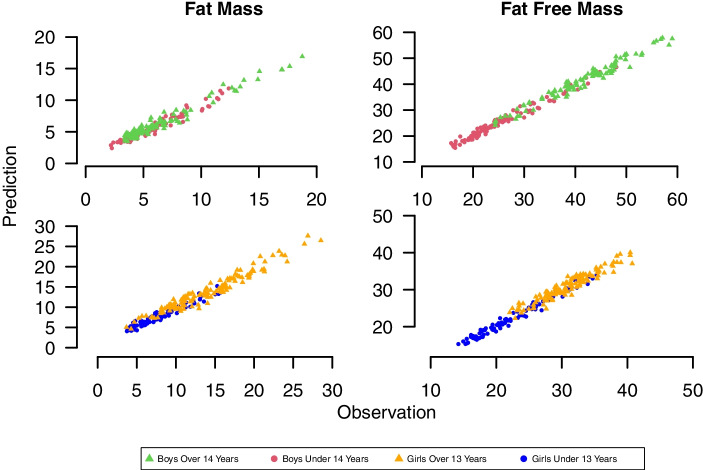
Quality of fit between fat-free mass and fat mass as derived by DXA with their counterparts predicted by the RGBN

The validity of RGBN performance to predict total fat mass and fat-free mass (lean and bone mass) is unknown. SEP values of less than 1.28 kg were found for boys and girls. The corresponding values were significantly high for all boys and girls. Fat mass could be estimated with a maximum difference of ± 9.98% of actual values. Additionally, the fat-free mass prediction had more accuracy than fat mass, with a maximum difference of ± 3.56% of actual values for boys and girls (Table 5). The predicted values of fat and fat-free mass versus the observed values, all of the correlations were significant (Fig. 4) (*P* < 0.001).

**Table 5:** Quality of fit between fat free mass and fat mass as derived by DXA with their counterparts predicted by the RGBN

	Fat Mass			Fat Free Mass		
	MAPE	SEP	R^2^	MAPE	SEP	R^2^
Boys over 14 years	9.98	1.10	0.94	3.39	1.74	0.95
Boys under 14 years	9.71	0.64	0.91	3.56	1.14	0.97
Girls over 13 years	8.69	1.27	0.91	3.29	1.24	0.90
Girls under 13 years	9.00	0.86	0.90	3.29	0.89	0.97

## Discussion

The importance of estimation of segmental body composition and finding interrelationships between them has been recognized by specialists and experts [38]. In the present cross-sectional study of Iranian children and adolescents, the segmental body composition components are estimated by using easy-to-measure anthropometric indices such as age, weight, height, BMI, arm circumference, and hip circumference, which have a high correlation with body composition components.

The trunk lean mass has been mentioned in the literature as an important measurement for overcoming musculoskeletal diseases [39, 40], as well as a critical supporting component of various spinal pathologies [41, 42]. Therefore, its evaluation in adolescence can improve the management and prevention of such diseases. To the best of our knowledge, the literature has focused more on predicting adolescents’ total fat-free mass [43] and anthropometric prediction equations were not available for predicting adolescents’ trunk lean mass. Accordingly, in the current study, this network method enables us to do this. Our findings revealed that the trunk lean mass had a highly significant association with boys’ height, weight, and hip circumference. Nevertheless, in girls, it was only significantly related to height and weight. Due to its causative role in several body composition components, the trunk lean mass is an essential variable in the hierarchical prediction approach, and some other segments’ estimation depends on its prediction.

Assessing appendicular (legs and arms) lean mass during early growth is especially important because of its association with health issues [44–49]. Maintaining optimal appendicular lean mass in adolescents may improve peak lean mass and bone strength, as well as have beneficial effects on adult cardiovascular health [50]. Moreover, low lean mass is associated with metabolic risk, and muscular strength is positively related to higher insulin sensitivity in children [51]. In our results, leg lean mass was associated with weight and height in boys, but in the age group under 14 years, it also had a significant relationship with hip circumference. In girls, leg lean mass was associated just with weight. The arm lean mass had a significant relationship with age in boys over 14 years old and with weight in girls over 13 years old. A cross-sectional study on adolescents found a high correlation with appendicular lean mass and weight, height, and age in both sexes and no mean differences between predicted and measured values [52].

A significant association was found between trunk fat mass and BMI and height in boys; in addition to BMI, this variable also had a significant relationship with weight in girls over 13 years of age and waist circumference in girls under 13 years of age. The literature review showed that BMI and waist circumference had a significant role in predicting fat mass [12, 15, 16]. A cross-sectional study in Brazilian adolescents reported that body circumferences, including waist circumference, had high adjusted coefficients of determination to estimate fat mass in girls [15]. Another study in schoolchildren and adolescents found that BMI and waist circumference are among the best predictors of body fat percentage [16].

About appendicular fat mass, leg fat mass was associated with age in boys over 14 years old and with height in girls over 13 years old. A significant relationship was found between leg fat mass and weight in both sexes in the younger age group. Also, arm fat mass had a significant association just with height in boys and girls. In both boys and girls, absolute and adjusted values of lean mass for body size increased with age, although to a higher amount in males. In teenagers, there was also a difference in body composition between the sexes [25]. This pattern of lean mass quantity and sexual dimorphism throughout adolescence is consistent with earlier results across ethnic groups and can be explained by hormonal effects. During pubertal growth spurts, growth hormone and sex steroids are dramatically activated, resulting in significant gains in skeletal muscle and, as a result, lean mass [53].

Regarding the relationship between body composition measurements, there was no significant association between segmental fat mass and BMC conditionally by segmental lean mass in any group. According to a research on Iranian children and adolescents, total body BMD and total body lean mass had the most correlation, whereas total body fat percentage had the least, and BMD distribution was affected by age and total body lean mass, which were also independent factors [54]. In another study on adolescents and young adults, whereas Pearson correlations between DXA measures of fat mass and bone parameters (BMC and BMD) were generally positive, the result showed that after accounting for lean mass and some other indices, fat mass did not correlate with DXA values for bone [55].

Based on these estimated body composition measurements, several indicators that are linked to human health concerns could be extracted, such as the body fat to lean mass ratio, which is a valuable metric for predicting and managing cardiometabolic events in the early stages [56]; the proportion of the trunk to appendicular fat mass increase is associated with an increase in blood pressure [57]; appendicular lean mass is an index to define sarcopenia, which is one component of malnutrition [45]; elevated values of this index may increase sensitivity to insulin [58] and development in bone health [46], in opposition, low values relate to metabolic risk factors and resistance to insulin [47, 48, 59], and including the risk of osteoporosis [49].

According to our findings, RGBN was an excellent way to predict segmental body composition compared to the multivariate regression model due to the considerable reduction in the parametric dimension and acceptable accuracy. Predictions using the present equations to estimate body composition components were reliable, given high coefficients of determination, which averaged 85% for boys and 79% for girls. The high correlation of body composition components from RGBN and DXA values (averaged R2: 0.87 for boys and 0.82 for girls) supports using RGBN equations to estimate body composition in children and adolescents.

These results are consistent with other studies based on the BN method that have indicated that anthropometric variables are good predictors of body components. L. Mioche et al. [22] used a non-parametric equation derived from a BN including sex, age, weight, and height to predict the fat-free mass. Their results indicated that correlations between BN predictions and DXA measurements are significant for fat-free mass. Furthermore, they confirmed that BN predictions are more accurate for fat-free mass and fat mass than those obtained from linear models. L. Mioche et al. [22] in another study assessed the robustness of the BN predictions and determined that predicted fat-free mass distributions are close to their published counterparts for both sexes between 20 and 79 years old; they suggested that, under certain anthropometric constraints, BN predictions might be used as a complementary body composition analysis for large populations.

An essential feature of our proposed model is that it can simultaneously predict several segmental compartments to better assess individuals’ health status or metabolic risks. To our knowledge, this is the first proposal for a network approach for the Iranian adolescent population. Although the direct method (DXA) and the estimation approach, including regression equations, Bayesian networks, or other appropriate methods based on anthropometric variables, in most cases show slight differences, the present equations are a safe alternative for implementation in non-clinical settings where there are no complex laboratories and equipment for evaluating body composition.

### Limitation

This study was limited in some aspects. The main limitation was its cross-sectional design. It may not be possible to generalize these findings to other populations. Furthermore, the results of the present study are based solely on a statistical model, and future prospective studies are required to accredit our observations. Biological plausibility has not been the objective of the present study and requires comprehensive discussion in another study.

## Conclusion

Based on the present study’s predictive models, adolescents' body composition might be estimated by using anthropometric measurements for a large population in non-clinical settings. Additionally, a few significant health risk factors that are involved with body composition components can be identified, such as excess fat mass, peak bone mass, and inadequate lean body mass of an adolescent. Given the flexibility and modeling of the present method to test different motivated hypotheses, its application to body compositional data and screening large populations is highly appealing.

## Data Availability

The datasets used and/or analyzed during the current study available from the corresponding author on reasonable request.

## Abbreviations

DXA: Dual-energy x-ray absorptiometry
BMI: Body mass index
UWW: Underwater weighing
ROC: Receiver operating characteristic
BN: Bayesian networks
RGBN: Robust gaussian bayesian network
BMC: Bone mineral content
BMD: Bone mineral density
TRD: Training dataset
GBN: Gaussian Bayesian network
SEP: Standard error prediction
MAPE: Mean absolute percentage error

## Footnotes

1 Since the present study was conducted on children, to avoid ambiguity, the word "offspring" was used instead of "child". In the context of Bayesian networks, child is a commonly-used word which means a node that is directly influenced by another node (called the parent).
